# Assessment of the Relation Between Diabetes Mellitus and Current Smokers Across Varying Demographics and Socioeconomic Characteristics: A Cross-Sectional Study

**DOI:** 10.7759/cureus.89269

**Published:** 2025-08-03

**Authors:** Shruti Suresh Suvarna, Shrishti Khetan, Dency D Mavani, Diya V Patel, Gayathri Dantu, Jahnavi Chagarlamudi, Deepthi Enumula

**Affiliations:** 1 Community Health Sciences, American University of Barbados, Wildey, BRB; 2 Internal Medicine, Smt. N. H. L. Municipal Medical College, Ahmedabad, IND; 3 Internal Medicine, Government Medical College, Mahabubnagar, IND; 4 Internal Medicine, Dr. D. Y. Patil Medical College, Hospital & Research Centre, Pune, IND; 5 Pharmacy Practice, Balaji Institute of Pharmaceutical Sciences, Warangal, IND; 6 Pharmacy Practice, Sri Ramachandra Institute of Higher Education and Research, Chennai, IND

**Keywords:** brfss, diabetes, odds ratio, retrospective study, smoking

## Abstract

Introduction: Smoking induces oxidative stress and inflammation, which can impair insulin function and increase the risk of developing diabetes. Understanding this modifiable risk factor across diverse demographic and socioeconomic groups is essential for targeted public health interventions.

Aims: To assess the relationship between current smoking and self-reported diabetes and to analyze how this association varies across demographics and socioeconomic characteristics.

Methodology: This retrospective study used data from the 2022 Behavioral Risk Factor Surveillance System (BRFSS). The primary variables were smoking status and self-reported diabetes. Control variables included age, gender, race, education, and income. Data was extracted using the BRFSS Web-Enabled Analysis Tool. Cross-tabulations were performed for each variable, with results reported as odds ratios (ORs) and 95% confidence intervals (CIs).

Results: A total of 408,857 respondents across all U.S. locations aged 18 years and older were recorded; among these, 7,028 (14.1%) current smokers reported having diabetes, while 49,652 (13.8%) non-smokers reported having diabetes. The likelihood of diabetes among current smokers was slightly higher but statistically non-significant (OR = 1.025, 95% CI: 0.997-1.053). Highest odds were observed in smokers aged 65+ (n = 2,522, 20.7%), female individuals (n = 3,603, 14.7%), non-Hispanic others (n = 1,381, 16%), and those with advanced education (n = 3,472, 13.4%). Lower odds were found in male individuals (n = 3,425, 13.6%) and Black, non-Hispanic (n = 830, 18.9%), low-income < $50,000 (n = 4,266, 16.5%), and basic education (n = 3,535, 14.9%). Therefore, the diabetes likelihood with smoking varies by age, sex, race, income, and education.

Conclusions: While the overall association between smoking and diabetes was statistically insignificant, notable differences were found among demographic and socioeconomic subgroups. Further prospective studies are recommended to explore these in depth.

## Introduction

Diabetes mellitus (DM) is a long-term metabolic condition marked by high plasma glucose levels. Regarding the disease's pathogenesis, two primary mechanisms have been identified. The autoimmune destruction of pancreatic β-cells leading to inadequate insulin production, along with the body's cells developing resistance to insulin, are the main contributors to the persistent hyperglycemia associated with DM [1]. In 2021, about 38.4 million people in the U.S., or 11.6% of the population, had diabetes [2]. Among adults, 14.7% were affected, with nearly a quarter of them unaware they had the condition. Prevalence increased with age, reaching 29.2% in those 65 and older [2]. Constant diet and glucose monitoring in diabetes impairs quality of life, causing distress, anxiety, non-compliance, and poor glucose control

Smoking is a major yet often overlooked risk factor for type 2 diabetes, contributing to insulin resistance and β-cell dysfunction, worsening glycemic control, mortality, and complications in diabetes. Its mechanisms are unclear; most studies have focused on nicotine, with limited investigation into other tobacco constituents [3], and smoking greatly elevates the likelihood of developing type 2 diabetes, with smokers having a 30%-40% higher chance of acquiring the disease compared to those who do not smoke. The presence of nicotine in tobacco can lead to increased blood sugar levels and inflammation, complicating the body's ability to manage blood sugar effectively [4].

This study has utilized the Centers for Disease Control and Prevention (CDC) 2022 Behavioral Risk Factor Surveillance System (BRFSS), a health survey at the national level that is conducted through telephone interviews, to look into the correlation between smoking and diabetes [5].

Aim and objectives

This study assesses the association between current smoking and diabetes prevalence in U.S. adults, adjusting for demographic and socioeconomic factors using BRFSS data.

## Materials and methods

This retrospective original research study was conducted using the BRFSS database. Data from this study was extracted on April 4, 2025, and analyzed. All the data from BRFSS were public, and this research qualifies as non-human subjects research, thus exempting the study from ethics committee approval.

Data were extracted using the BRFSS Web-Enabled Analysis Tool (WEAT) for the year 2022. The primary variable of interest in this study was the disease variable Diabetes, “Ever told you had diabetes excluding pregnancy (DIABETE4),” and the lifestyle risk factor variable smoking, “Calculated variable for adults who are current smokers (_RFSMOK3).” The study included several control variables to adjust for potential confounders, ensuring a comprehensive analysis. Demographic characteristics were carefully considered, including age (_AGE_G: 18-24, 25-44, 45-64, 65+), gender (respondents' sex, categorized as male and female), and race (calculated variable for four race groups, categorized as White (non-Hispanic), Black (non-Hispanic), and Hispanic/other races). Socioeconomic characteristics included education level (EDUCA), ranging from never attended school to college graduates, and annual income (_INCOMG_), categorized as <$15,000 and >$200,000, graded by seven levels. Participants who responded to the selected disease, risk factor, and control variables were included in the study, ensuring completeness of data for analysis. Data from those who responded to the above questions were included in this study, as these are the variables that were selected for the study.

Participants with incomplete or missing responses for any of the selected variables, including disease status, smoking status, age, gender, race, education level, or income level, were excluded from the study. Respondents who refused to answer or selected “Don’t know/Not sure” for any of these variables were also excluded. Additionally, individuals under 18 years of age were not considered, as the BRFSS survey includes only adults aged 18 years and older.

Descriptive statistics, including numbers and percentages, were generated for each variable using cross-tabulations in the BRFSS WEAT. The data were exported to Microsoft Excel (Microsoft Corp., Redmond, WA) for organization and further processing. Statistical analyses were conducted to assess associations between categorical variables using R software, version 4.3.1 (R Foundation for Statistical Computing, Vienna, Austria, https://www.r-project.org/). Results were expressed as odds ratios (ORs) with 95% confidence intervals (CIs). A p-value of <0.05 was considered statistically significant.

## Results

In the year 2022, the BRFSS recorded a total of 408,857 respondents across all U.S. locations aged 18 years and older. Among these, data on current smoking status (a modifiable lifestyle factor) and self-reported diabetes (ICD-10: E10-E14) were analyzed. The final analysis included all records (n = 408,857), with no exclusions. Among these, 7,028 (14.1%) current smokers reported having diabetes, while 49,652 (13.8%) non-smokers reported diabetes. The odds ratio (OR) for diabetes among current smokers was 1.025 (95% CI: 0.997, 1.053), suggesting a marginal and statistically nonsignificant increased likelihood of diabetes in this group, as represented in Table 1.

**Table 1:** Prevalence of current smokers in participants with self-reported diabetes *Significant; CI: confidence interval; OR: odds ratio

Current smokers	Yes	Diabetes		OR (CI)
		Yes	No	1.025 (0.997, 1.053)*
		7028 (14.1%)	42750 (85.9%)	
	No	49652 (13.8%)	309427 (86.2%)	

Temporal trends and age-adjusted mortality rates could not be evaluated within this cross-sectional dataset, as BRFSS is a point-in-time survey without follow-up years. However, stratified prevalence across age groups revealed significant trends, with younger smokers (18-24 years) showing higher odds of diabetes (OR: 1.788, CI: 1.224, 2.544), decreasing with age until 65+, where the odds became protective (OR: 0.949, CI: 0.906, 0.993), suggesting age-specific risk profiles. Among the total current smokers analyzed, males accounted for 25,208 (13.6%) diabetic cases and females for 24,570 (14.7%). Interestingly, female current smokers exhibited a higher likelihood of diabetes compared to their male counterparts, with an OR of 1.146 (CI: 1.103, 1.19) in female individuals and 0.91 (CI: 0.876, 0.946) in male individuals, indicating a demographic disparity in the gender-based risk. Racial distribution showed the highest proportion of diabetes among current smokers was in non-Hispanic Whites (n = 4,630, 13.2%), followed by non-Hispanic Blacks (n = 830, 18.9%) and non-Hispanic other races (n = 1,381, 16%). The highest diabetes risk was observed in the non-Hispanic Other group (OR: 1.16, CI: 1.089, 1.234), followed by non-Hispanic Blacks (OR: 0.913, CI: 0.84, 0.99), suggesting race-based disparities in diabetes prevalence among smokers, as shown in Table 2.

**Table 2:** Prevalence of current smokers in participants with self-reported diabetes based on demographic characteristics *Significant; CI: confidence interval; OR: odds ratio

Demographic characteristics	Current smokers	N	Participants with diabetes	Participants without diabetes	OR (CI)
Age groups					
18-24 years	Yes	1606	36 (2.2%)	1570 (97.8%)	1.788 (1.224, 2.544)*
	No	23223	294 (1.3%)	22929 (98.7%)	
25-44 years	Yes	14597	836 (5.7%)	13761 (94.3%)	1.546 (1.427, 1.672)*
	No	83528	3159 (3.8%)	80369 (96.2%)	
45-64 years	Yes	21363	3634 (17%)	17729 (83%)	1.212 (1.165, 1.26)*
	No	115042	16644 (14.5%)	98398 (85.5%)	
65+ years	Yes	12212	2522 (20.7%)	9690 (79.3%)	0.949 (0.906, 0.993)*
	No	137286	29555 (21.5%)	107731 (78.5%)	
Gender					
Male	Yes	25208	3425 (13.6%)	21783 (86.4%)	0.91 (0.876, 0.946)*
	No	167867	24720 (14.7%)	143147 (85.3%)	
Female	Yes	24570	3603 (14.7%)	20967 (85.3%)	1.146 (1.103, 1.19)*
	No	191212	24932 (13%)	166280 (87%)	
Race					
White, non-Hispanic	Yes	35207	4630 (13.2%)	30577 (86.8%)	1.008 (0.975, 1.042)*
	No	262484	34286 (13.1%)	228198 (86.9%)	
Black, non-Hispanic	Yes	4402	830 (18.9%)	3572 (81.1%)	0.913 (0.84, 0.99)*
	No	26968	5473 (20.3%)	21495 (79.7%)	
Non-Hispanic/other race	Yes	8619	1381 (16%)	7238 (84%)	1.16 (1.089, 1.234)*
	No	59531	8409 (14.1%)	51122 (85.9%)	

While geographic characteristics such as urbanization and place of death were not applicable in this survey-based dataset, the prevalence was compared based on socio-economic factors. A higher prevalence of diabetes was observed in current smokers with basic education (n = 3,535, 14.9%) than in those with advanced education (n = 3,472, 13.4%). Interestingly, the odds of diabetes were lower in current smokers with basic education (OR: 0.802, CI: 0.771, 0.834) but significantly higher in those with advanced education (OR: 1.107, CI: 1.066, 1.15), reflecting a potential inverse socio-educational trend. In terms of income, individuals earning less than $50,000 per year had higher diabetes prevalence among current smokers (n = 4,266, 16.5%) compared to those earning more than $50,000 (n = 1,716, 10.5%). The odds of diabetes in lower-income smokers were significantly lower (OR: 0.833, CI: 0.804, 0.864) than in non-smokers in the same income group, while the higher-income group showed no significant association (OR: 1.006, CI: 0.954, 1.06), as represented in Table 3.

**Table 3:** Prevalence of current smokers in participants with self-reported diabetes based on socio-economic characteristics *Significant; CI: confidence interval, OR: odds ratio

Socioeconomic characteristics	Current smokers	N	Participants with diabetes	Participants without diabetes	OR (CI)
Education level					
Basic education	Yes	23786	3535 (14.9%)	20251 (85.1%)	0.802 (0.771, 0.834)*
	No	98350	17579 (17.9%)	80771 (82.1%)	
Advanced education	Yes	25838	3472 (13.4%)	22366 (86.6%)	1.107 (1.066, 1.15)*
	No	259311	31881 (12.3%)	227430 87.7%)	
Annual income					
Less than $50,000	Yes	25850	4266 (16.5%)	21584 (83.5%)	0.833 (0.804, 0.864)*
	No	112209	21513 (19.2%)	90696 (80.8%)	
Greater than $50,000	Yes	16375	1716 (10.5%)	14659 (89.5%)	1.006 (0.954, 1.06)*
	No	179896	18748 (10.4%)	161148 (89.6%)	

## Discussion

This retrospective study analyzed data from the 2022 CDC BRFSS to determine the relationship between current smoking status and self-reported current diabetes among the US population of people aged 18 years and above. Drawing a total of 408,857 samples with no record being excluded in the study, the emphases were placed on the demographic and socioeconomic covariates of the relationship. Current smokers had a prevalence rate of 14.1% for diabetes compared to 13.8% among non-smokers, with the odds ratio (OR) being 1.025 (CI: 0.997, 1.053). Despite this non-significant overall result, the data revealed significant disparities according to age, gender, race, education, and income levels. Notably, female smokers, young smokers within 18-44 years, and non-Hispanic others had significantly higher odds ratios with diabetes. Among socioeconomic characteristics, smokers with advanced education had higher odds of diabetes, and lower-income smokers (<$50,000) also showed elevated risk.

Smoking is associated with diabetes in both physiological and epidemiological aspects. Smoking has been associated with insulin resistance, inflammation, oxidative stress, and abdominal obesity [6], which are defined to contribute to type 2 diabetes. Nicotine hampers glucose uptake and hinders the functioning of beta cells, thus raising the chances of hyperglycemia and diabetes [7]. A study showed that smokers have higher chances of developing type 2 diabetes than non-smokers [8]. Smoking is recognized as a modifiable behavioral risk factor in diabetes, with Durlach et al. (2022) stating that smoking contributes to the development of diabetes and aggravates diabetes complications [9].

This study revealed an overall OR of 1.025 with diabetes in current smokers, suggesting a modest but notable association in line with existing literature. In contrast to the previous works [10] that suggested a strong relationship of smoking with diabetes in younger age groups, this study registered evidential importance only among specific subpopulations. This disparity could partly be explained by increased health literacy [11], better access to diabetes checkups, and smoking cessation campaigns that have been embarked on in the recent past. Alternatively, the BRFSS’s reliance on self-reported data could lead to underreporting of both smoking and diabetes, potentially diluting observed associations.

Demographic disparities in morbidity were evident. Female smokers had a higher likelihood of diabetes as opposed to male smokers (OR: 1.146 vs. OR: 0.91), opposing some studies that pointed towards a greater cardiovascular and metabolic disease risk among male smokers [12]. This could be explained by biological predisposition, differences in utilization of health care, or higher prevalence of psychosocial stress in women. Racial differences were also observed, with smokers of non-Hispanic and other ethnicity having higher odds of diabetes (OR=1.16), and Black and non-Hispanic smokers having slightly lesser odds (OR=0.913). These disparities may be due to structural inequalities, cultural beliefs, genetic predispositions, and health literacy [13]. Eastwood et al. (2024) add evidence to these findings by highlighting the complex interaction of race, lifestyle, and health outcomes [14].

Geographic disparities were also observed. Although specific mortality data were not stratified by urbanization in this dataset, there is a general trend seen [15] that individuals in nonmetropolitan or rural settings have higher risks for chronic conditions and lower access to preventive services. This is due to the reduced healthcare accessibility, low income, and higher rates of smoking among the rural dwellers. These patterns corroborate the study by Kobo et al. (2022), which revealed the increased rural-to-urban differential in mortality and morbidity ratio due to diabetes [16].

Temporal trends, though not directly measured in this cross-sectional study, remain essential to contextualizing our findings. The persistent or rising odds of diabetes in certain smoking subgroups suggest that public health measures have not uniformly benefited all populations. Gender- and race-based differences underscore the need for targeted interventions. Although smoking rates are declining in the general population, smoking prevalence remains disproportionately high among low-income and minority groups, sustaining health inequities [17].

The observed associations and disparities emphasize the urgent need for tailored public health strategies. Future research should explore the effects of smoking cessation programs stratified by demographic and socioeconomic status, evaluate access to diabetes screening and education, and assess policy-level interventions. Efforts to reduce diabetes prevalence must include behavioral risk factor modifications, equitable healthcare access, and culturally competent health promotion across diverse populations [18].

Limitations

This study has several limitations. As a retrospective and observational analysis of BRFSS 2022 data, it cannot establish causality. Self-reported information may introduce recall or reporting bias. The dataset lacks clinical validation and excludes variables such as comorbidities, healthcare access, and treatment history, which may confound associations. Additionally, socioeconomic and lifestyle factors may influence outcomes but were not fully captured, potentially impacting the accuracy of observed relationships.

## Conclusions

This study analyzed data from the 2022 BRFSS to explore the relationship between current smoking and self-reported diabetes among U.S. adults aged 18 and above. Although the overall association was modest and statistically nonsignificant, subgroup analyses revealed important disparities. Elevated diabetes prevalence among female smokers, young adults, and the non-Hispanic "other" racial/ethnic group, alongside the contrasting impacts of lower income and higher education, highlight the varied nature of this relationship. These variations likely stem from structural inequities, differential access to healthcare, varying health literacy, and the impact of targeted health messages. Consequently, public health interventions must move beyond generalized approaches to embrace focused, equitable strategies tailored to high-risk demographics. Future research, particularly longitudinal studies, should delve deeper into the complex interplay of smoking, diabetes risk, and socioeconomic factors to inform more effective and culturally sensitive prevention and cessation efforts.
